# Self-Healing Poly(urea formaldehyde) Microcapsules: Synthesis and Characterization

**DOI:** 10.3390/polym15071668

**Published:** 2023-03-27

**Authors:** Jehan Kothari, Jude O. Iroh

**Affiliations:** Mechanical and Materials Engineering Department, University of Cincinnati, Cincinnati, OH 45221, USA

**Keywords:** self-healing, microcapsules, poly(urea formaldehyde), epoxy, diisocyanates, glass transition temperature

## Abstract

Smart coatings and smart polymers have been garnering great interest in recent times due to their novel characteristics, such as being self-restoring, self-cleaning, and self-healing. However, most self-healing materials have a low glass transition temperature (T_g_) and are inadequate for the repair of advanced composites. Because of their low T_g_, the conventional self-healing materials plasticize and weaken the composites. In this study, moderate to high temperature self-healing microcapsules, capable of healing and thus stopping crack propagation, are prepared. The microcapsules were prepared using a two-step process involving the synthesis of poly(urea formaldehyde) (PUF) prepolymer, followed by the encapsulation of hexamethylene diisocyanate (HDI) in an oil-in-water emulsion to form a crosslinked PUF shell. Diisocyanates are of particular interest as self-healing encapsulants because of their diversity of structure and fast rate of hydrolysis. Successful encapsulation was verified by Fourier transform infrared spectroscopy (FTIR) and optical microscopy. Thermogravimetric analysis (TGA) was used to characterize the thermal properties of microcapsules. The onset temperature for microcapsule degradation varied from 155 °C to 195 °C. Dynamic mechanical analysis (DMA) was used to determine the thermomechanical response of microcapsule/epoxy films. DMA showed that the glass transition temperature (T_g_) of the epoxy/microcapsule composite was greater than the T_g_ for neat epoxy and varied between 34 and 65 °C. The TGA analysis of the epoxy/microcapsule composite shows that the thermal stability and char retention of the epoxy/microcapsule composite increased and the low temperature decomposition peak at 150 °C, associated with the microcapsule, disappeared after the DMA test, indicating the occurrence of a reaction between HDI and the epoxy to form a crosslinked polyurea network structure.

## 1. Introduction

**Scheme 1:**
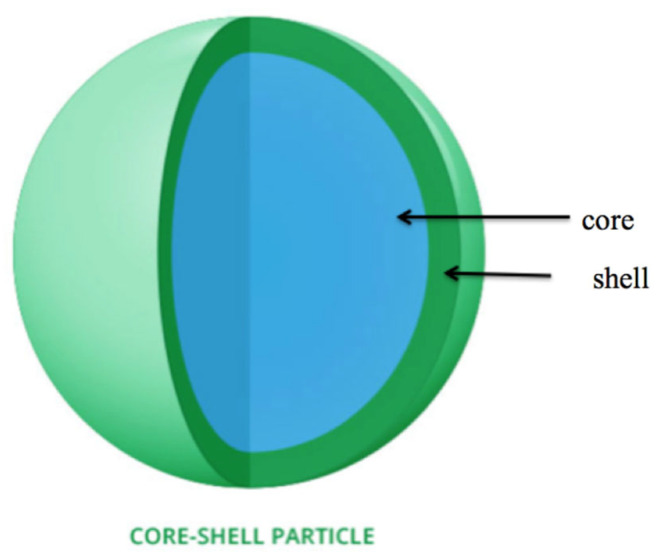
Illustration of a microcapsule.

**Scheme 2:**
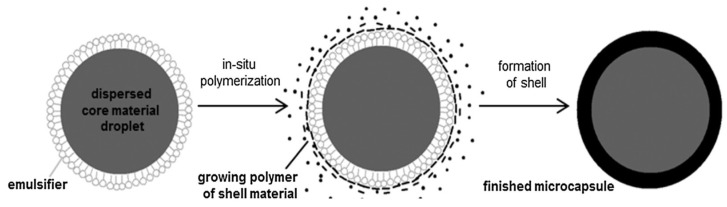
Mechanism of microcapsule formation.

A microcapsule consists of a shell and a core [1] (Scheme 1 and Scheme 2). The shell is the protective cover for the core, which is the active agent. In the case of self-healing systems, the core is a readily polymerizable material that can heal the damaged area when the microcapsule ruptures and the core is released.

A microcapsule is formed in an emulsion in which the core is the dispersed phase, while the shell-forming material is integrated into the continuous phase. As most of the core materials are insoluble in water, an oil-in-water emulsion facilitates the formation of the microcapsules. The shell material starts polymerizing on the interface of the core and continuous, and on the completion of the polymerization forms a hard crosslinked protective shell (Scheme 2).

The study of the synthesis and analysis of self-healing polymers has intensified in recent years [2,3,4,5,6,7,8,9,10,11,12,13,14,15,16,17]. Brown et al. studied the effect of microcapsule concentration, catalyst concentration, and healing time on the efficiency of the self-healing system [2,3]. The authors used a modified tapered double cantilever beam (TDCB) to measure the healing efficiency of the self-healing system. The healing efficiency was measured as η = Pc_healed_/Pc_virgin_, where Pc_healed_ is the critical fracture load of the specimen healed after rupture and Pc_virgin_ is the critical fracture load of the virgin specimen. The authors showed that as the catalyst content increased the healing efficiency increased and the gel time decreased. In order to balance the gel time, catalyst concentration was restricted to 2.5%. The concentration of the microcapsules had a similar effect on the healing efficiency. The healing efficiency increased with the increase in microcapsule concentration, but at the same time, sample preparation became harder due to the increased viscosity. The authors suggested that a concentration of up to 15% was feasible for processing and high healing efficiency. A maximum healing efficiency of greater than 90% was recorded by the authors [2].

The authors went on to synthesize DCPD encapsulated in polyurea formaldehyde in an oil and water emulsion. The average diameter of the microcapsule was between 10–1000 µm. It was observed that the average diameter decreased with the increased agitation rates. Microcapsules had a shell thickness between 160 and 220 nm. It was observed that during microcapsule synthesis, urea formaldehyde adhered to the microcapsules, producing a rough exterior morphology which, in turn, increased the adhesion of microcapsules to the epoxy matrix. To decrease the adhesion, the interface between water and DCPD was increased. A fill content of 83–92 weight percent was recorded by the authors [3].

Yuan et al. synthesized PUF microcapsules encapsulating epoxy resin. They employed a two pot system for microcapsule synthesis. The authors prepared a PUF prepolymer in the first step. In the next step, a solution of the prepolymer, along with an emulsifier, was made in water. To this solution, a mixture of diglycidyl ether of bisphenol A (DGEBPA) and 1- butyl glycidyl ether (BGE) as a reactive diluent was added to form an oil-in-water emulsion. The pH was decreased, and the reaction was conducted at 60–65 °C for 3 h to obtain epoxy-encapsulated PUF microcapsules. The authors found that the mixture of DGEBA and BGE reacts negligibly with NH linkages in PUF as the reactivity between the NH groups and epoxide ring is negligible below 200 °C. The core-to-shell ratio affected the size of the microcapsule. An increase in the ratio caused an increase in the size of the microcapsule due to the increase in size of the DGEBA and BGE droplets in the emulsion during synthesis [4]. They also studied the thermal effect on the microcapsules [4] and found that the chemical stability of their system lasted until 200 °C, while the physical stability of the system was maintained up to 180 °C. They observed that the microcapsule deformation due to heat increased as the shell wall thickness decreased and as the roughness of the microcapsule, due to PUF nanoparticles, increased. They also reported that the deformation of microcapsules below 251 °C was due to the diffusion of the core, while that above 251 °C was due to the decomposition of the shell wall and crosslinking. Thus, thermal stability was recorded to be approximately 180 °C [5].

The authors further studied the effect of surfactant on the microcapsules. They noted that the particle size of the microcapsules decreased as the surfactant concentration increased. An increase in the rate of heating increased the diameter of the microcapsule slightly. The time given for the pH adjustment had a slight effect on the system too. A decrease in time, given for pH adjustment, increased the size of the microcapsules. The optimum process parameters for the system were found to be a SDBS concentration of 1 weight %, heating rate of 0.2 °C/min, and a pH adjusting time of 2–3 h [6].

As a continuation of their previous work, Yuan et al. worked on the encapsulation of polythiol, used as a curing agent for epoxies, which can be later used as a two-part curing system with encapsulated epoxies. The authors used poly(melamine-formaldehyde) (PMF) as the encapsulating material instead of PUF, as it takes a shorter time to form. This is especially helpful for polythiol, as they have a high reactivity. PMF microcapsules also show higher mechanical properties when compared with PUF microcapsules. A similar two pot method was employed in which they synthesized PMF oligomer in the first step. In the second step, they created an oil-in-water emulsion with the polythiol, pentaerythritol tetrakis (3-mercaptopropionate) (PETMP) as the oil phase, and the PMF oligomer in the continuous phase. The authors were able to improve the synthesis procedure by optimizing the process parameters and found 40–60 min at 50 °C at a pH of 2.9–3.2 as the optimal process parameters [7].

Wu et al. [7,8] prepared microcapsules with a polyurea/silica hybrid shell and hexamethylene diisocyanate as the core, forming a one part self-healing system [8,9]. Unlike two-part systems of epoxy and hardener or epoxy and catalyst, proposed by the various researchers mentioned above, a one part self-healing system is economically more viable and has a greater potential for mass production as the need for a microencapsulated hardener or catalyst impregnation within the matrix is eliminated. The microencapsulation process utilized a combination of two strategies, interfacial polymerization and an in-situ sol-gel process carried out in an oil-in-water emulsion.

The encapsulation procedure for this method is a one-pot process. The aqueous solution of gum arabic along with HDI and MDI prepolymer (Suprasec 2644) formed the oil-in-water emulsion. The emulsion was then heated at 40 °C and agitated to emulsify the mixture of MDI and HDI. After the emulsification, polyethylenimine (PEI) was added dropwise to the stabilized emulsion to form the polyurea shell. The amino groups in PEI tend to react with the isocyanate groups of the MDI prepolymer rather than HDI, as MDI has a higher reactivity. This encapsulated HDI in the hence formed PU shell. Tetraethyl orthosilicate (TEOS) was used as the precursor for silica moieties.

It was necessary to pre-hydrolyze TEOS in hydrochloric acid (HCL) of pH 2.2 as the rate of hydrolysis of TEOS is higher than the condensation rate of TEOS near a pH of 2, which is the isoelectric point of silica. Hydrolyzed silica was then added to the mixture, which got attracted to the PU shell by means of hydrogen and electrostatic attraction between the amino groups of the PU shell and silanol groups of TEOS. In order to increase the rate of condensation of TEOS, ammonia water was added to increase the pH, in the end forming a polyurea/silica hybrid shell around the HDI core.

The authors reported average diameters of 57–328 µm for the microcapsules for RPM ranging from 300 to 1000. They also observed that as the RPM increased, the core fraction of the microcapsules decreased due to the higher exposure of HDI to water that led to its consumption. TGA results showed a retention of 86.5% after exposure at 60 °C for 12 h. The authors conducted a solvent resistance test by the immersion of microcapsules in xylene for 100 h and obtained a value of 25.9 ± 0.7 wt%, showcasing good solvent resistance. The authors also conducted a rudimentary corrosion test in which they immersed a control and a specimen coated with the epoxy/microcapsule system in 10-wt% NaCl for 48 h. They observed rusting on the control, while the epoxy/microcapsule system showed no signs of rusting [8].

Wu et al. also worked on creating a superhydrophobic, self-cleaning, and self-healing system, consisting of a PUF shell and HDI core. The microcapsule synthesis was a two-step process. In the first step, the authors reacted urea and formalin at a basic pH to form the UF prepolymer. In the second step, the authors created an oil-in-water emulsion. A solution consisting of DI water, resorcinol, and prepolymer formed the aqueous phase. The solution was agitated, and the HDI was added dropwise to form the emulsion. The pH was decreased, and the UF microcapsules encapsulating the HDI were achieved. The solvent resistance of the HDI microcapsules was tested by immersion in various solvents for 16 days. They showed a final value of 4.13 ± 0.12 wt% in hexane, 3.13 ± 0.12 wt% in xylene, and 10.13 ± 0.12 wt% in chloroform. The onset of weight loss in the TGA analysis for HDI in the microcapsules was found to be 250 °C. The authors encapsulated 1H,1H, 2H, 2H perflurooctyltriethoxysilane (POTS), wax, HDI dimer, and xylene besides HDI. HDI and POTS containing microcapsules displayed superhydrophobicity on aging, while those containing xylene and wax did not. The authors concluded that the reactive cores of POTS and HDI core leach out of the shell, creating a secondary shell on the outer side on the microcapsule due to their moisture sensitivity, which leads to the hydrolysis of POTS and HDI [9].

Jin et al. synthesized a self-healing system comprising of a dual wall PU/UF microcapsule, encapsulating an epoxy resin, DGEBA, with a reactive diluent to decrease the viscosity, o-cresyl glycidyl ether (o-CGE). PUF microcapsules were used to encapsulate the hardener for the epoxy resin, namely, polyoxypropylenetriamine (POPTA). The dual wall microcapsule for the epoxy resin and its diluent was prepared using the method described previously by Caruso et al. [10]. The encapsulation of the hardener is particularly challenging by the standard methods of encapsulation as the amine functionality is highly reactive. Mcllroy et al. prepared amine microcapsules via the interfacial polymerization of the amine and an isocyanate in an inverse Pickering emulsion, but they were able to achieve a fill content of only 55% [11]. Jin et al. employed the vacuum infiltration of POPTA into hollow shells of polyurea formaldehyde for encapsulation [12].

The hollow microcapsules were prepared in two steps. Urea formaldehyde prepolymer was synthesized in the first step. In the second step, the prepolymer along with deionized water and an emulsifier were added to a beaker and agitated to form air bubbles. The entrapped air formed the core, and PUF polycondensation took place around it forming hollow microcapsules.

The authors reported a virgin fracture toughness recovery of 90% for epoxy matrix cured at 121 °C. The healing efficiency diminished as the temperature of curing for the epoxy matrix with the microcapsules increased. This was because as the temperature increased, the diffusion of the core materials through the shell also increased. The optimal ratio of microcapsules encapsulated with epoxy to microcapsules encapsulated with POPTA was found to be 1:1 [12].

Common applications of self-healing polymers are in the repair of fractured and deformed polymers, as well as in corrosion inhibition and corrosion sensing. Polymeric materials, such as epoxy esters, polyurea, polydimethylsiloxane, and polyimides and their copolymers, have been the main focus [12,13,14]. Copolymerization and blending are key strategies in designing a self-healing polymer system. The properties of epoxy ester resins are significantly improved by copolymerization with polyurea and polysiloxane [15,16,17]. High temperature self-healing materials can be formed by the copolymerization of polyurea and polyimide. Such a copolymer is known to possess high T_g_, of approximately 218 °C, and excellent durability [18,19,20]. Usually, the hydrophobic polyurea unit is inserted into the polyimide backbone to form a robust block copolymer.

Polyimide–polyurea copolymer coating with a remarkable lifetime, and outstanding corrosion inhibition properties have been reported [19].

Corrosion inhibitors can be released after deformation or fracture to form an anti-diffusion thin coating over the damaged region. Though chromium compounds are effective corrosion-inhibiting materials because of their ability to slow crack growth, they are considered to be environmentally unsafe [21]. The addition of microcapsules can improve the corrosion-inhibition properties while maintaining environmental friendliness. Microcapsules containing corrosion-inhibiting anions can be released, to displace and replace chloride ions [22], and form a micropores-coating complex. The microcapsule trapped in the coating must have a controlled release for the desired period of deployment [23,24]. Capsules containing self-healing compounds can be dispersed throughout a polymer matrix, so that the deformation or fracture of the polymer may trigger deployment and self-healing via polymerization and crosslinking [25,26,27]. A major advantage for the use of microcapsules with self-healing capabilities include their small size scale, requiring minimal material usage. It is noted that self-healing is a two-step process in which the microcapsules are initially deployed to form a passive thin film that provides a barrier against corrosion; subsequently, the polymer, after thermal annealing, can produce the shape memory response to heal the deformation or fracture [28,29,30].

In this study, the effect of repeated DMA scans and the prolonged aging of epoxy/microcapsule composites on the thermal and dynamic mechanical properties of the composite were determined. This study seeks to demonstrate the self-healing effect by analyzing changes in the T_g_ of an epoxy/microcapsule composite after repeated DMA scans in tensile mode. TGA analysis as well as FTIR spectroscopy of the samples after repeated DMA tests and prolonged time aging were used to collaborate DMA results.

## 2. Experimental

### 2.1. Materials

**Scheme 3:**
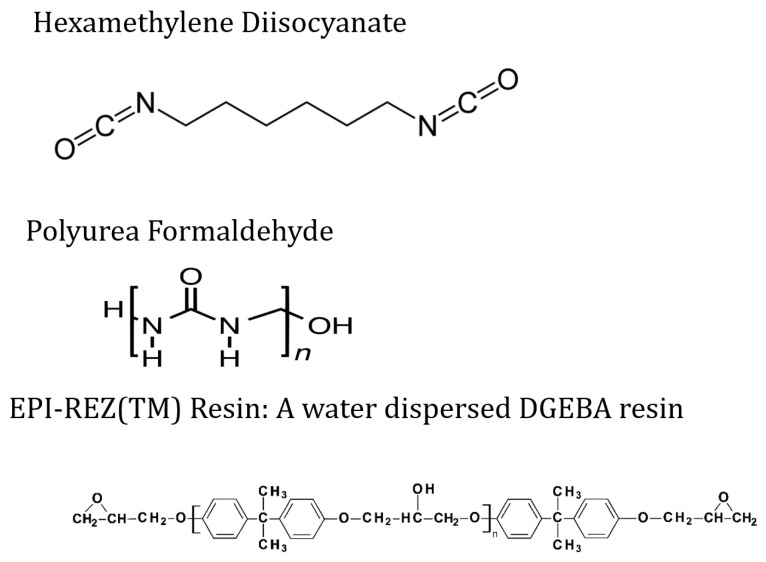
Chemical structure of materials.

The raw materials used in synthesizing the urea formaldehyde prepolymer were urea and a formaldehyde aqueous solution (37%). The synthesis required a pH adjustment, and this was done using citric acid (aqueous solution was pH 0.8), and 1 molar aqueous solution of NaOH, both of which were prepared in the lab. The raw materials required for the emulsion preparation were ethylene maleic anhydride (EMA) (2.5 weight percent aqueous solution), resorcinol, and hexamethylene diisocyanate. All the raw materials mentioned above were obtained from Sigma-Aldrich and were used as obtained. To make epoxy films filled with microcapsules, EPIREZ 5522 WY 55 epoxy (Scheme 3) dispersion along with EPIKURE 3234 aliphatic amine curing agent were used. The resin and curing agent were obtained from Hexion and were used as obtained.

### 2.2. Synthesis of Urea Formaldehyde Prepolymer

**Scheme 4:**
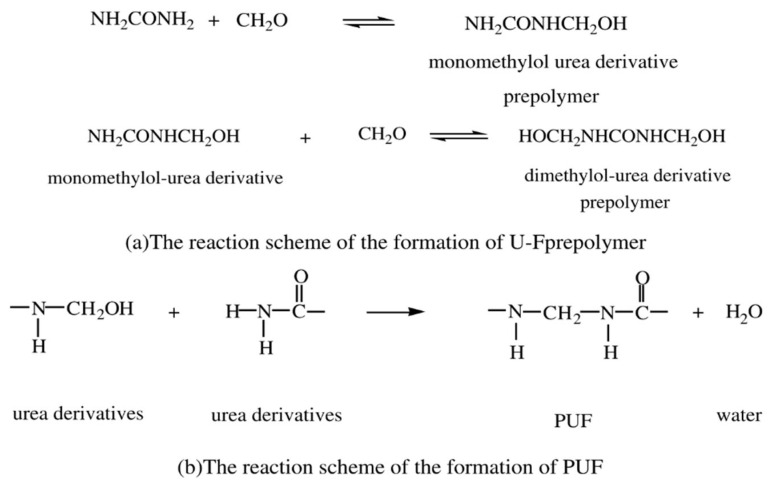
Representation of structures of polyurea formaldehyde, PUF [4,5].

**Scheme 5:**
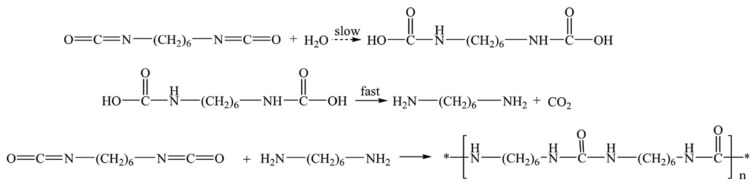
Representation of synthesis of polyurea based on HDI and MDI.

The first step in the preparation of the microcapsules was the synthesis of the urea formaldehyde prepolymer. In a typical process for the synthesis of the prepolymer, 2.5 g of urea and 5.96 g of aqueous formaldehyde were taken to ensure that the urea:formaldehyde ratio was 0.66 or a theoretical free formaldehyde percentage of 20%. The pH of aqueous formaldehyde was adjusted in the range of 8–9 with 1 molar solution of aqueous NaOH. Urea was added after pH was achieved. After achieving a reaction temperature of 70 °C, over a period of 30 min, the reaction mixture was allowed to react for another hour under continuous magnetic stirring. After an hour, the urea formaldehyde prepolymer was obtained and was brought back to room temperature by cooling in a water bath (Scheme 4 and Scheme 5). The measured final pH for all prepolymer was recorded in the range of pH 6 to pH 7.

### 2.3. Preparation of Microcapsules

The second step in the preparation of the microcapsules includes the formation of the oil-in-water emulsion. For this step, 0.23 g of resorcinol, 2.5 weight percent aqueous EMA solution (0.5% of the total amount of reactants), and deionized water (depending on the water:oil ratio desired) were added to a flask along with the prepolymer previously synthesized. The mixture was allowed to stir at an RPM of 200 for ten minutes. This formed the continuous phase of the emulsion. The RPM was then increased to 600, and 9.2 g of HDI was added dropwise to the aqueous solution to create an oil-in-water emulsion. The mixture was emulsified at 600 RPM for 20 min. The pH of the system was then adjusted to 1.55 using an aqueous solution of citric acid (pH 0.8). The reaction mixture was allowed to mix for another 30 min at room temperature after which the reaction temperature was increased to 55 °C over a period of 30 min. It was reacted at 55 °C for 2 h, after which microcapsules were obtained. The microcapsules were rinsed with DI water and acetone and were then filtered by means of vacuum filtration. An analysis of the microcapsules was performed after they were air dried for 24 h.

### 2.4. Preparation of Epoxy Film Filled with Microcapsules

Reacting the resin and curing agent created epoxy films. A ratio of 4:1 of resin-to-curing agent was used to produce virgin films. A typical procedure for creating the films was as follows: 4 g of epoxy resin and 1 g of curing agent were taken in a 20 mL flask. The resin and curing agent were diluted with 1 g DI water to lower the viscosity. A total of 0.5 g of the microcapsules were added to the system, followed by further dilution with 1 g DI water. The system was stirred with the help of a magnetic stirrer for 2 min. Pouring the mixture onto a glass slide formed epoxy films. The mixture was reacted in an oven at 60 °C for 2 h, and then allowed to further react at room temperature for 48 h before carrying out further tests.

### 2.5. Characterization

Fourier transform infrared spectroscopy (FTIR) was performed by using the Thermo Scientific Nicolet 6700 spectrometer, purchased from Thermo Scientific Company, Waltham, MA, USA in order to determine the chemical structure of the polymers. Approximately 32 scans were performed in the wavenumber range from 4000 to 400 cm^−1^. FTIR analysis was also used to determine the characteristic functional groups present in the microcapsules.

The dynamic mechanical analysis (DMA) was performed by using Seiko Instruments, SII EXSTAR 6000 Spectrometer, operated in the tensile mode, to determine the dynamic mechanical behavior of the microcapsules. It was purchased from Seiko Instruments company in Indianapolis, Indiana, USA. Thin films of approximately 20 mm (L) × 8 mm (W) × 0.8 mm (t) were tested. DMA tests were performed in a temperature range between −100 °C and 200 °C at a heating rate of 5 °C/min and a frequency of 1 Hz. The data obtained from the DMA test include the glass transition temperature (T_g_), the melting temperature (T_m_), the storage modulus (E′), the loss modulus (E″), and the damping behavior measured in terms of tan δ peak height and peak area for the α-transition.

Thermogravimetric analysis (TGA) was used to determine the thermal stability and decomposition of the samples. The samples were tested at the heating rates of 10 °C/min in a temperature range between 25 and 600 °C in an inert nitrogen atmosphere. The TGA test was performed by using the Q50 Thermal Analyzer purchased from TA Instruments, New Castle, DE, USA.

## 3. Results and Discussion

### 3.1. FTIR Analysis

**Figure 1:**
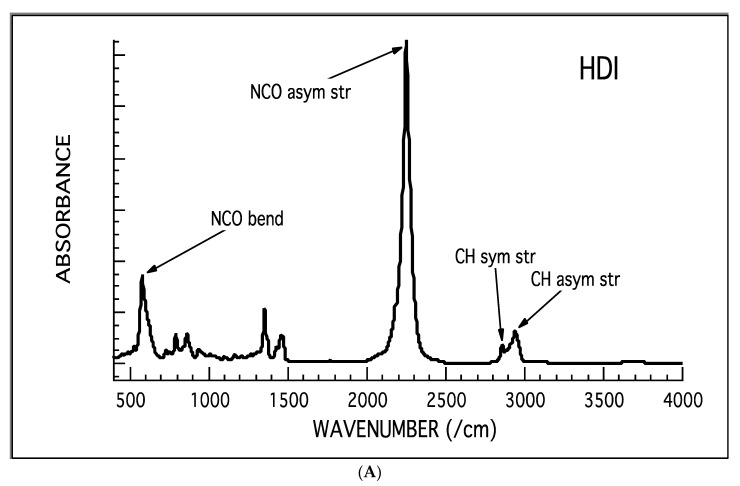

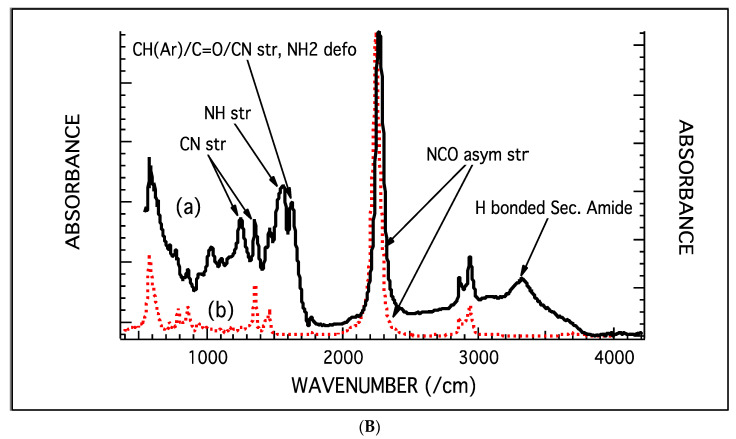
(**A**) FTIR of HDI. (**B**) FTIR of microcapsules with 22% free formaldehyde shell:core ratio 1:2 and water:oil ratio of 5.5:1. FTIR of: (a) Microcapsules with a water-to-oil ratio of 5.5:1; (b) HDI.

Figure 1A,B show the FTIR spectra for HDI (Figure 1A) and that for the microcapsule (Figure 1B). The FTIR spectrum for HDI showed strong signal peaks at 589 cm^−1^ relating to the NCO bending vibration, at 2250 cm^−1^ relating to the NCO stretching, and at 2866 cm^−1^ and 2936 cm^−1^ relating to the CH symmetric and asymmetric stretching. The FTIR spectrum of the microcapsule, prepared by using a water-to-oil ratio of 5.5:1 shows the characteristic absorption peak due to CH stretching which is overlapped with the peak due to OH stretching to form a broad peak at 3198 cm^−1^. The FTIR peak at 1610 cm^−1^ is due to the conjugated aryl carbon–carbon double bond, C=C stretching. The absorption peak at 1373 cm^−1^ is due to the CH in plane bending vibration. The C–OH stretching vibration doublets are found at 1373 cm^−1^ and 1297 cm^−1^ and at 1147 cm^−1^ and 1167 cm^−1^. The benzene ring vibrations due to =C–H and C=C stretching are found at 1489 cm^−1^ and 1607 cm^−1^, and finally, the out of plane deformation due to four neighboring hydrogen atoms of meta di-substituted ring are located at 843 cm^−1^ and 739 cm^−1^.

**Figure 2:**
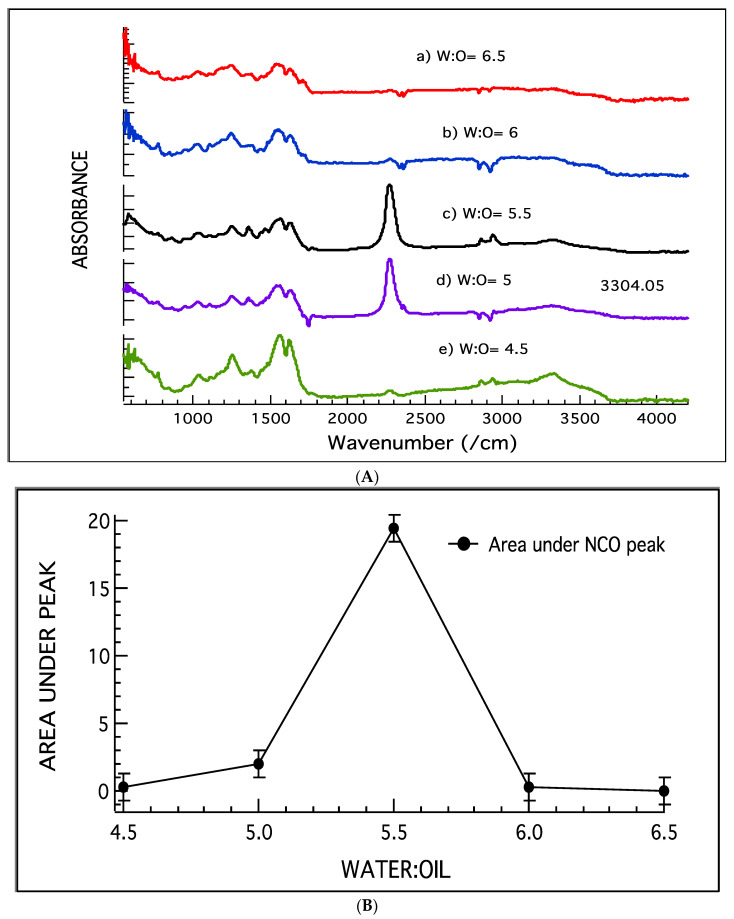
(**A**) FTIR of samples with water-to-oil ratio of 4.5:1 to 6.5:1. (**B**) The area under NCO peak as a function of water-to-oil ratio used in synthesis of microcapsules.

The FTIR spectra for the microcapsules prepared by using a water-to-oil ratio of 4.5:1, 5:1, 5.5:1, 6:1, and 6.5:1 are shown in Figure 2A. The FTIR peaks due to the hydrogen-bonded secondary amide group are shown at 3321 cm^−1^, 3342 cm^−1^, 3322 cm^−1^, and 3356 cm^−1^. The FTIR peaks at 1554 cm^−1^, 1555 cm^−1^, 1550 cm^−1^, and 1548 cm^−1^ are due to NH stretching. Peaks due to CN stretching are shown at 1359 cm^−1^ and 1252 cm^−1^ for microcapsules, MCs with a water-to-oil ratio of 4.5:1, and 1359 cm^−1^ and at 1260 cm^−1^ for MCs with a water-to-oil ratio of 5:1, 1355 cm^−1^ and 1252 cm^−1^ for MCs with a water-to-oil ratio of 5.5:1, and 1360 cm^−1^ and 1244 cm^−1^ for MCs with a water-to-oil ratio of 6:1. Peaks due to C=O stretching are shown at 1630 cm^−1^, 1634 cm^−1^, 1630 cm^−1^, and 1638 cm^−1^ for samples with a water-to-oil ratio of 4.5:1, 5:1, 5.5:1, and 6:1, respectively. The presence of a secondary amide group, CN and C=O, suggests the existence of a PUF shell. Resorcinol shows a weak peak due to the four neighboring hydrogen atoms of the Meta di-substituted ring at 733 cm^−1^, 726 cm^−1^, 721 cm^−1^, and 735 cm^−1^ for samples with a water-to-oil ratio of 4.5:1, 5:1, 5.5:1, and 6:1, respectively. The peak of most significance was the peak due to NCO stretching which is shown at 2274 cm^−1^, 2272 cm^−1^, 2274 cm^−1^, and 2271 cm^−1^ for samples with a water-to-oil ratio of 4.5:1, 5:1, 5.5:1, and 6:1, respectively. The NCO stretching peak indicates the encapsulation of HDI.

#### Microcapsules with a Water-to-Oil Ratio of 6.5:1

Microcapsules with a water-to-oil ratio of 6.5:1 showed a negligible NCO peak, thus confirming that encapsulation did not take place. The presence of CH symmetric stretching, CH asymmetric stretching, NH deformation, CN stretching, and NH stretching at 2849 cm^−1^, 2917 cm^−1^, 1498 cm^−1^, 1600 cm^−1^, and 3220 cm^−1^, respectively, showed the presence of polyurea and PUF.

Figure 2B shows the area under the NCO peak of microcapsules with water-to-oil ratios of 4.5:1, 5:1, 5.5:1, 6:1 and 6.5:1. From Figure 2B, it is clear that the amount of NCO content and thus the amount of HDI was highest in the sample with a water-to-oil ratio of 5.5:1.

### 3.2. TGA Analysis

**Figure 3:**
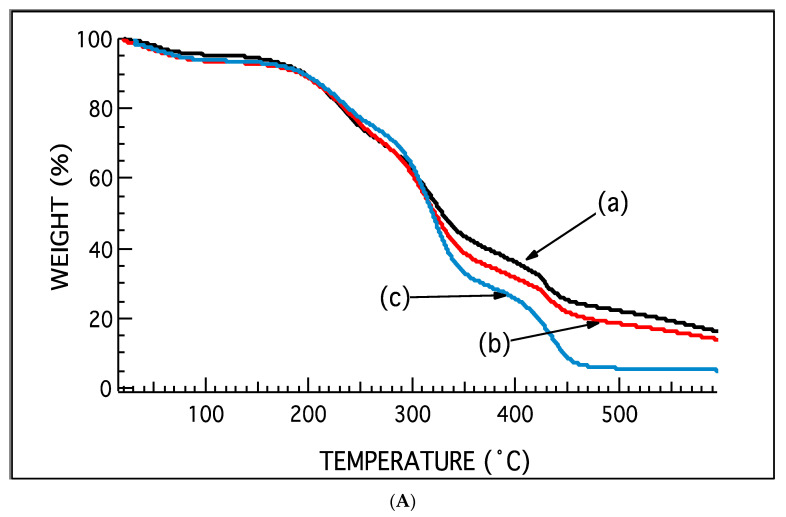

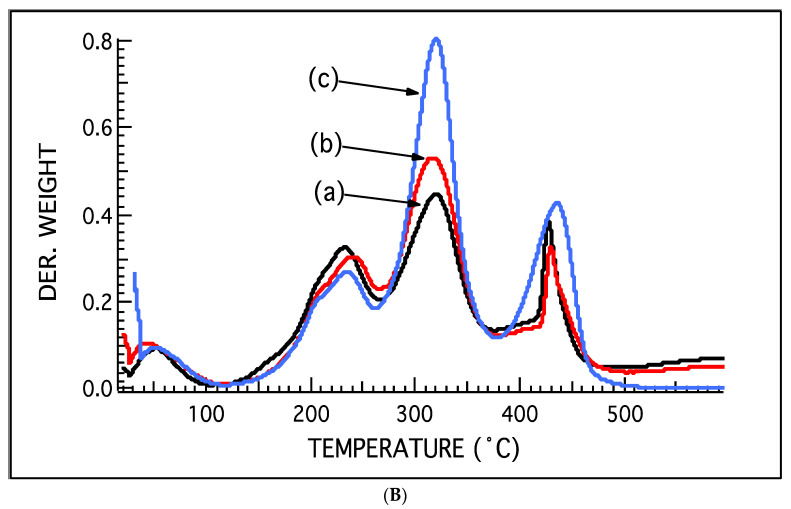
(**A**) Comparison of percent weight as a function of temperature for microcapsules with 22% free formaldehyde shell:core ratio 1:2 and varying water:oil ratios. (a) W:O = 4.5:1; (b) W:O = 5:1; (c) W:O = 5.5:1. (**B**) Comparison of derived weight as a function of temperature for microcapsules with 22% free formaldehyde shell:core ratio 1:2 and varying water:oil ratios. (a) W:O = 4.5:1; (b) W:O = 5:1; (c) W:O = 5.5:1.

**Figure 4:**
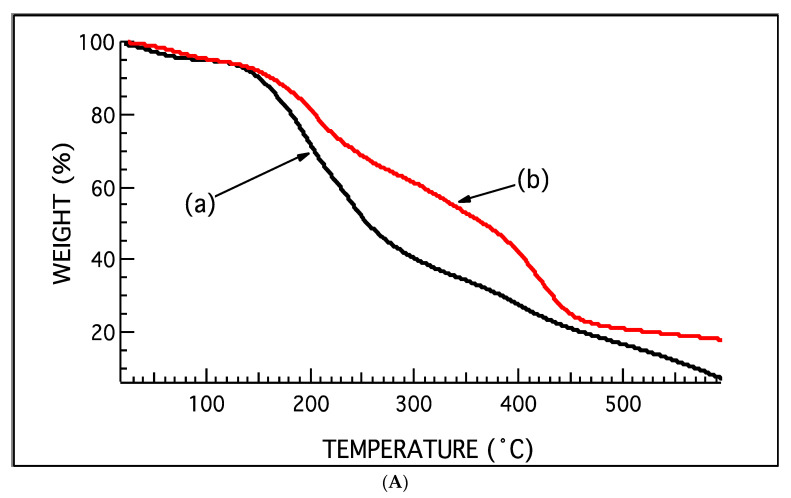

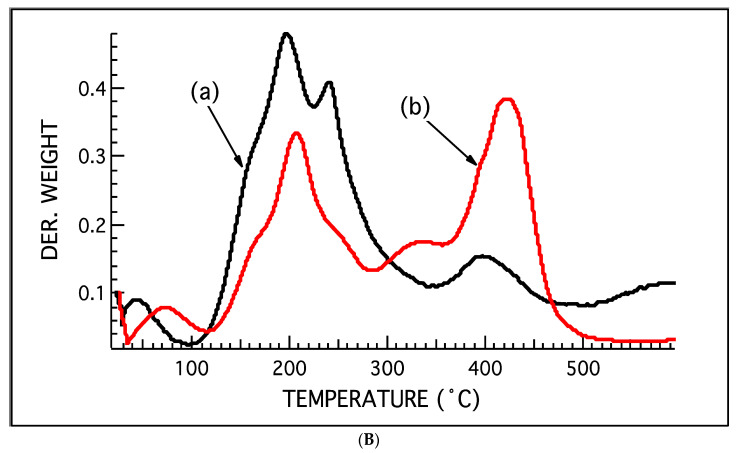
(**A**) Comparison of percent weight as a function of temperature for microcapsules with 22% free formaldehyde shell:core ratio 1:2 and varying water:oil ratios. (a) W:O = 6:1; (b) W:O = 6.5:1. (**B**) Comparison of derived weight as a function of temperature for microcapsules with 22% free formaldehyde shell:core ratio 1:2 and varying water:oil ratios. (a) W:O = 6:1; (b) W:O = 6.5:1.

The TGA analysis of the microcapsules shows four distinct transitions regions as seen in the individual graphs of samples prepared with varying water:oil ratios ranging from 4.5:1 to 6.5:1 (Figure 3 and Figure 4). The first transitions region is below 100 °C and can be attributed to the evaporation of water and free formaldehyde. The second transition region is shown from approximately 155 °C to 195 °C. It signifies the degradation of the PUF shell. The third transition region occurs in the temperature range between 236 °C and 307 °C, which coincides with the boiling point of HDI (255 °C) (Figure 3A,B). It is therefore due to the evaporation of HDI from the core. The fourth transition region is located beyond 350 °C, and it is due to the degradation of polyurea formed and is associated with the hydrolysis of HDI when the shell of microcapsules ruptures. The transition region observed at the elevated temperature range is probably due to the fact that the PUF shell protected the HDI.

The weight loss in transition 3 relating to HDI is highest in the microcapsules with a water:oil content of 5.5:1, followed by 5:1, 4.5:1, and 6:1, and the least amount of weight loss was found in the sample with a water-to-oil ratio of 6.5:1. On the other hand, the highest weight loss for transition 4, relating to polyurea formation due to shell rupture, was found in 6.5:1, followed by 5.5:1, 4.5:1, and 5:1, and the least was found in 6:1. Microcapsules with a water-to-oil ratio of 6:1 showed a fifth transition region (Figure 4A,B). The fifth transition region may have arisen due to the interaction of the core with the shell. The microcapsules with a water-to-oil content of 6.5:1 have the least weight loss due to HDI and the highest weight loss due to polyurea formation. It can be concluded that the majority of microcapsules in this sample broke after encapsulation. At the same time, microcapsules with a water-to-oil content of 6:1 had the second lowest percentage weight loss due to HDI and the lowest percentage weight loss due to polyurea, suggesting that encapsulation took place to an extremely low extent in this sample. It can also be noted that this sample had the highest percentage weight loss due to PUF and thus had a very high content of PUF nanoparticles. Since microcapsules with a water-to-oil content of 5.5:1, 5:1, and 4.5:1 had the highest weight loss due to HDI, these samples were the most efficient in encapsulating HDI.

#### Yield

**Figure 5:**
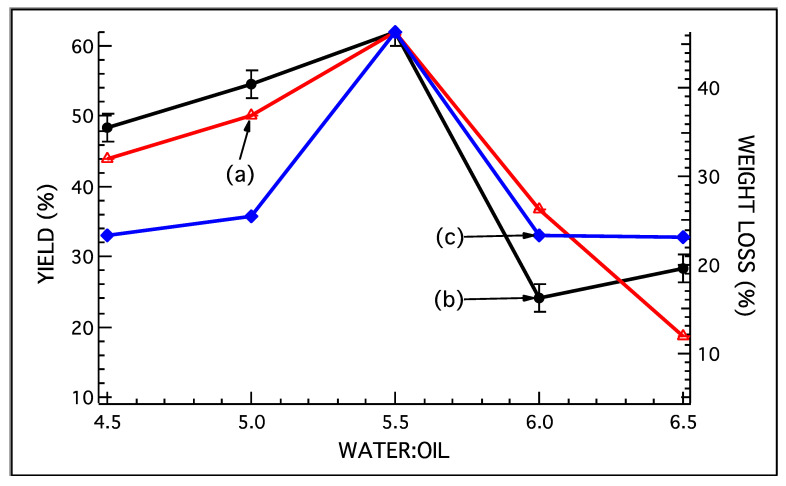
Percentage of weight loss due to HDI (a), yield (b), and area under the NCO peak from FTIR (c) as a function of the water-to-oil ratio.

The yield was calculated gravimetrically with the help of a sensitive weight balance. It was simply taken as the percentage of the microcapsule weight after filtration and drying to the raw material weight. Maximum yield was obtained in the sample with a water-to-oil ratio of 5.5:1 (Figure 5). The yield increases until a water-to-oil ratio of 5.5:1 and then decreases again. A similar trend was observed for weight loss in transition 3, relating to HDI in TGA, and the area under the NCO peak calculated in FTIR. The increase in yield with an increasing amount water is well documented by several authors, including Brown et al. and Yuan et al. [2,3]. The prepolymer of urea formaldehyde is soluble in water. As the amount of water increases, the prepolymer gets diluted. The precipitation of the prepolymer onto the dispersed phase of the HDI depends on the concentration of the prepolymer. Higher polymer concentrations precipitate at a faster rate than in lower concentrations [12]. HDI is also water sensitive, and the lower rate of shell formation might have affected the yield above a water-to-oil ratio of 5.5:1, as HDI might have been consumed by the aqueous phase.

### 3.3. Optical Microscopy

**Figure 6:**
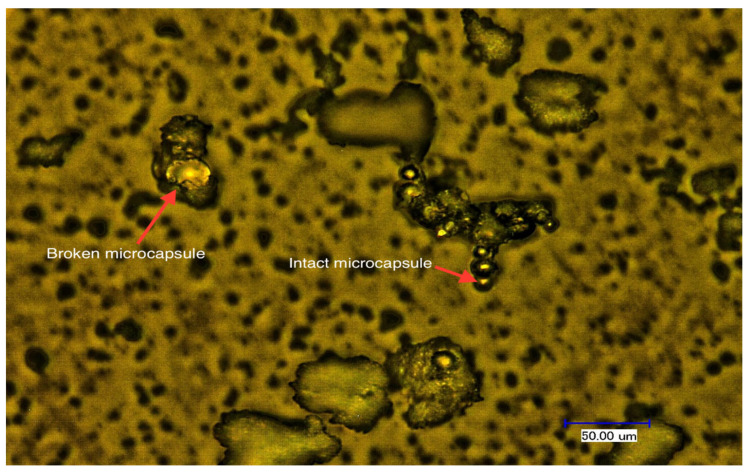
Optical image of a microcapsule with a water:oil ratio of 5.5:1.

The size of the microcapsules was calculated as a mean of 100 such microcapsules and was found to be 17 microns for microcapsules with water as 5.5 (Figure 6). The encapsulation can be confirmed by the presence of a diffraction ring. Light travels through the solid shell into the liquid core and out again through the solid shell. The difference in the refractive index of the shell and core creates the diffraction ring.

### 3.4. DMA Analysis

For the purpose of DMA, test specimens were prepared by adding self-healing microcapsules at a concentration of 10% of the epoxy resin used. It was then cured at a room temperature for 2 days and at 60 °C for 2 h. DMA was carried out at a heating rate of 5 °C per minute. The specimen was cooled below freezing point to −50 °C and then heated to 150 °C under oscillating stress to carry out DMA testing. The DMA curves encompass the loss modulus, storage modulus, and the tan∂ curves. The T_g_ of the system can be calculated from tan∂ or the loss modulus.

**Figure 7:**
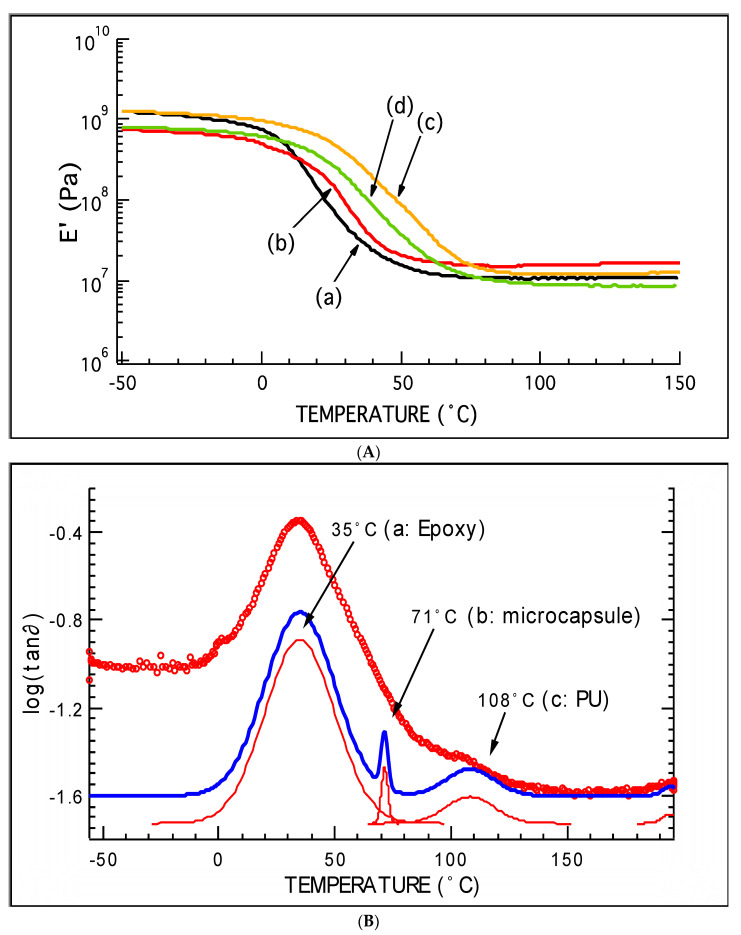

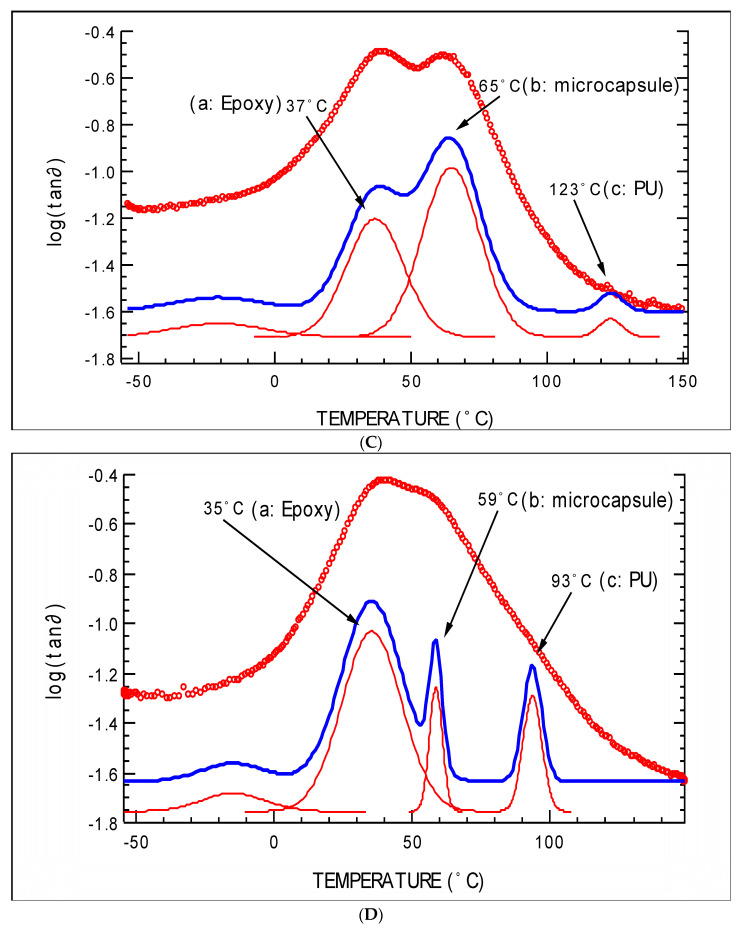
(**A**) Comparison of the storage modulus of pure epoxy with epoxy filled with microcapsules synthesized using varying water:oil ratios. Storage modulus of: (a) pure epoxy; (b) W:O = 4.5:1; (c) W:O = 5:1; (d) W:O = 5.5:1. (**B**) Gaussian fitting of log (tan(∂)) of epoxy filled with microcapsules synthesized using a water:oil ratio of 4.5:1. Peak due to: (a) epoxy; (b) microcapsule; (c) PU/epoxy interaction. (**C**) Gaussian fitting of log (tan(∂)) of epoxy filled with microcapsules synthesized using a water:oil ratio of 5:1. Peak due to: (a) epoxy; (b) microcapsule; (c) PU/epoxy interaction. (**D**) Gaussian fitting of log (tan(∂)) of epoxy filled with microcapsules synthesized using a water:oil ratio of 5.5:1. Peak due to: (a) epoxy; (b) microcapsule; (c) PU/epoxy interaction.

For pure epoxy, the T_g_ is approximately 27 °C (Figure 7). Tan∂ peaks for the epoxies containing microcapsules showed a broad peak, which when integrated resolved into three peaks. To find the T_g_ from tan∂, Gaussian curve fitting was performed using IGOR, and the fit peaks gave the T_g_ of the system. As shown in Figure 7A,B, epoxy filled with microcapsules synthesized with a water-to-oil ratio of 4.5:1 showed a compound peak consisting of one major peak at 35 °C and two shoulders. The most prominent peak at 34.9 °C can be attributed to epoxy resin. The increase in T_g_ from the virgin epoxy can be attributed to the particulate composite formed due to the addition of the microcapsules. The peak at 71.5 °C is sharp and might be attributed to smaller chains or monomers, and a broad shoulder was observed at 108.2 °C (Figure 7B). The broad shoulder at 108.2 °C might be due to interaction between the polyurea formed by rupture of the microcapsule and consecutive hydrolysis of HDI and the epoxy matrix. The smaller sharper peak might be due to microcapsules that have not ruptured and contain HDI. Similar broad tan∂ peaks were observed in epoxy filled with microcapsules synthesized with a water-to-oil ratio of 5:1 and 5.5:1. The tan δ curve for the specimen containing microcapsules synthesized with a water-to-oil ratio of 5:1 (Figure 7C), two distinct peaks were formed at 36.82 °C and 64.74 °C, as seen in Figure 7C. Unlike the peak at 71.5 °C, observed for the previous sample, the peak at 64.74 °C is broader and much more prominent. The peak separation into an individual peak might suggest the phase segregation of microcapsules and epoxy. The shoulder at 123.27 °C was also smaller. According to the previous assumption, this might be indicative of lesser interactions between epoxy and polyurea. For the specimen containing microcapsules synthesized with a water-to-oil ratio of 5.5:1, the peak corresponding to epoxy and microcapsules was found more or less at the same temperatures that were observed in previous specimens (Figure 7D). The peak relating to epoxy is observed at 35.39 °C, the one relating to microcapsules at 58.58 °C, and that of the PU/epoxy interaction is found at 93.49 °C. The nature of the interaction is beyond the scope of this study, and further characterization would be required to know the same.

**Figure 8:**
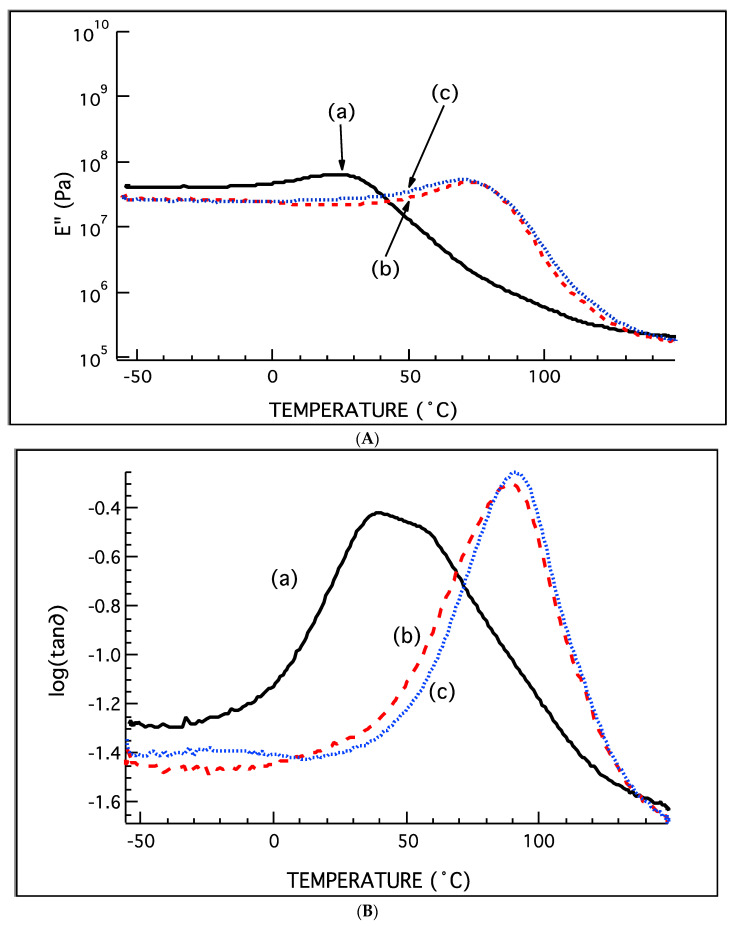
(**A**) Comparison of the loss modulus for epoxy filled with microcapsules synthesized using a water:oil ratio of 5.5:1 and run for three cycles: (a) first cycle; (b) second cycle; (c) third cycle. (**B**) Comparison of log (tan(∂)) for epoxy filled with microcapsules synthesized using a water:oil ratio of 5.5:1 and run for three cycles: (a) first cycle; (b) second cycle; (c) third cycle.

A multi-cycle DMA was conducted for an epoxy/microcapsule system with a water-to-oil ratio of 5.5:1 (Figure 8A,B). The broad tan δ peak was resolved into three peaks (Figure 8B), but this peak vanishes and became a single peak at the end of the third run (Figure 8A,B). The area under the tan δ curve is related to the chain relaxation behavior of PU/epoxy interactions, and the temperature corresponding to the peak is associated with the glass transition temperature, T_g_. The T_g_ increases in every step and remains at an almost constant temperature after the second and third traces. The resolution of the broad compound peak to a single peak also signifies that the system moved towards a single homogeneous phase. The rupture and cure of HDI led to a more continuous phase, which resulted in a single peak.

**Figure 9:**
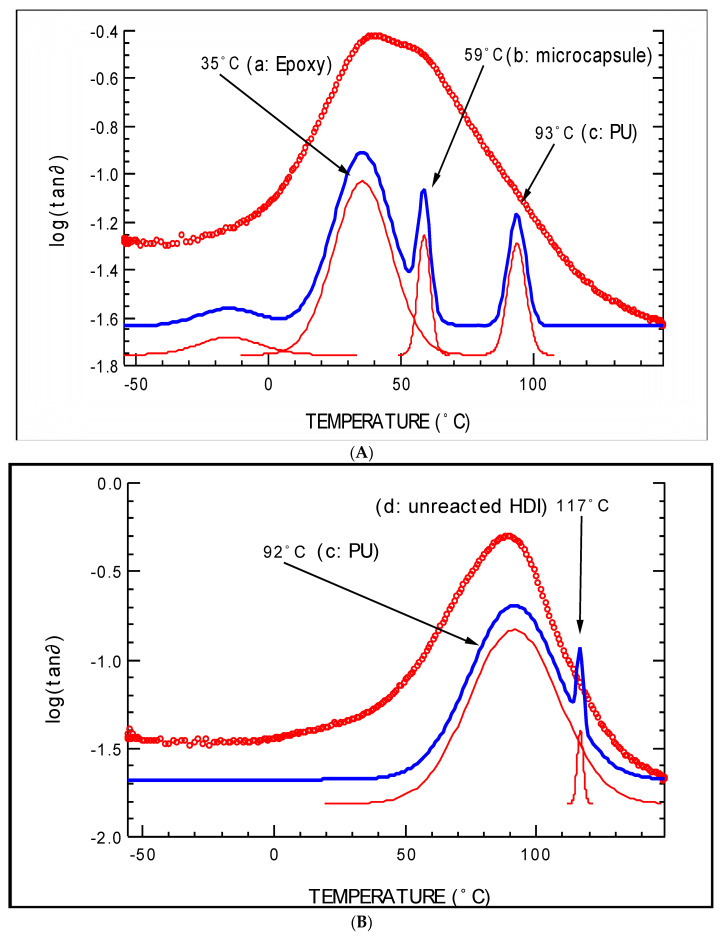

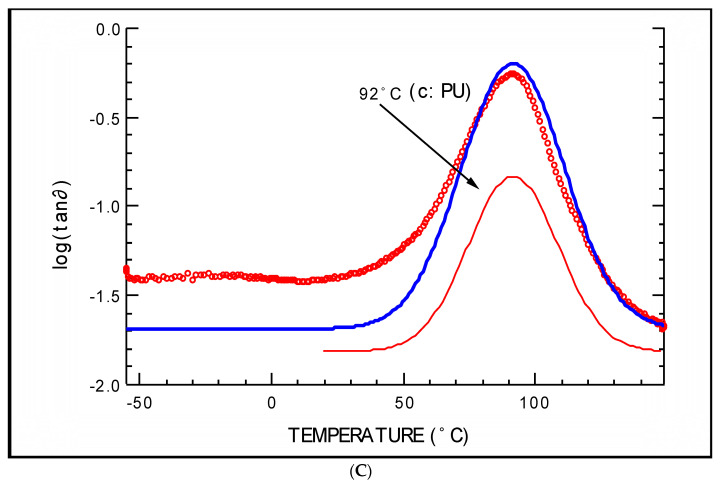
(**A**): Gaussian fitting of log (tan(∂)) of epoxy filled with microcapsules synthesized using a water:oil ratio of 5.5:1 (step 1). Peak due to: (a) epoxy; (b) microcapsule; (c) PU/epoxy interaction. (**B**) Gaussian fitting of log (tan(∂)) of epoxy filled with microcapsules synthesized using a water:oil ratio of 5.5:1 (step 2). Peak due to: (c) PU/Epoxy interaction; (d) unreacted HDI. (**C**): Gaussian fitting of log (tan(∂)) of epoxy filled with microcapsules synthesized using a water:oil ratio of 5.5:1 (step 3). Peak due to: (c) PU/epoxy interaction.

Figure 9A–C shows the Gaussian fitting and deconvolution of the tan δ versus temperature peaks for the repeated (three) cycles of dynamic mechanical testing. The Gaussian fitting of the broad peak obtained from the first cycle shows three major peaks at 35 °C for epoxy resin, another peak at 59 °C for the microcapsules, and a third peak at 93 °C for PU (Figure 9A and Table 1).

The Gaussian fitting of the tan δ peak obtained from the second run shows only two peaks (Figure 9B). The peak at 35 °C has disappeared and the predominant peak occurs at 92 °C for PU. A sharp and narrow peak is shown at 117 °C and is believed to be due to unreacted HDI (Figure 9B).

Gaussian fitting of the tan δ versus temperature peak obtained from the third DMA scan of the same sample (Figure 9C), shows only one major peak at 92 °C due to polyurethane, PU. Analysis of the data obtained from these repeated runs suggests that the reaction of epoxy and HDI to form PU is completed after the third cycle, resulting in a homogenous PU phase. The intermediate products formed after the first and second cycles (repeated tests) were converted into PU during the third cycle or repeated testing.

### 3.5. TGA Analysis

**Figure 10:**
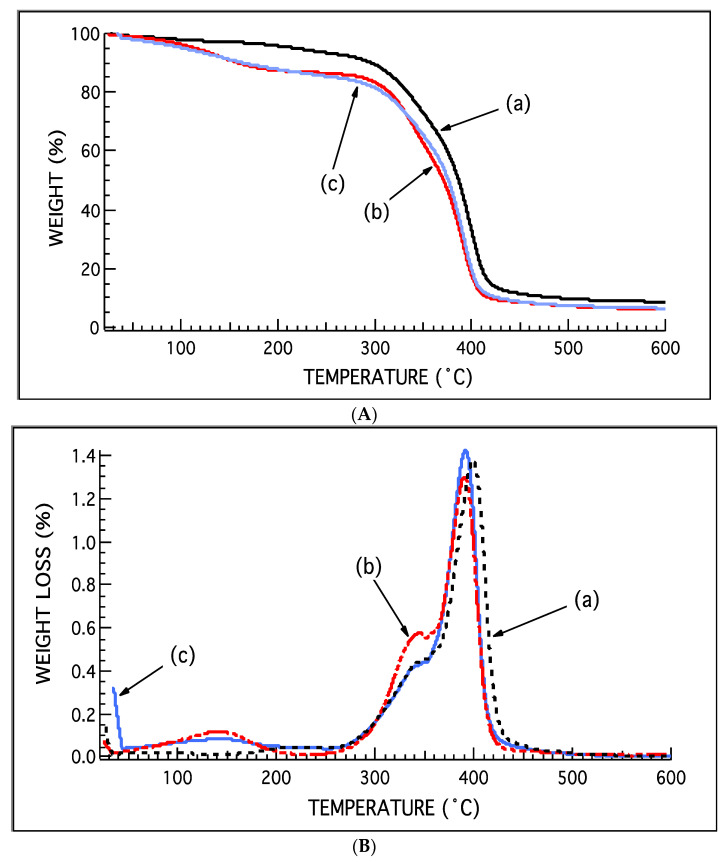
(**A**) Comparison of the TGA weight loss of: (a) epoxy/microcapsule composite after DMA test; (b) neat epoxy; (c) epoxy/microcapsule composite. (**B**) Comparison of derived weight of: (a) epoxy/microcapsule composite after DMA; (b) neat epoxy; (c) epoxy/microcapsule composite.

The TGA traces of neat epoxy and epoxy filed with microcapsules are shown in Figure 10A,B. The derivative TGA trace (DTGA) for the samples (Figure 10B) show three decomposition peaks at 140 °C, 340 °C, and 380 °C. The DTGA, the TGA of epoxy/microcapsule obtained after the DMA test, showed three decomposition peaks at 350 °C and 400 °C. The broad and weak peak at 140 °C disappeared after the DMA scan, but the temperature for the major weight loss at 380 °C shifted to a higher temperature of 400 °C (Figure 10B). A very weak and broad peak can be seen between 180 °C and 250 °C, and it is believed to be due to the rupture of the PUF during the DMA test. TGA decomposition peaks 2 and 3 make for the maximum weight loss in neat epoxy and epoxy/microcapsule composites before and after the DMA test. These peaks are attributed to TETA and DGEBA linkages. The degradation temperature for the microcapsules/epoxy system and that for neat epoxy are very similar. The amount of the microcapsules loaded into the epoxy is 10%. Thus, the individual decomposition peaks for the microcapsules do not appear in the epoxy/microcapsule composite TGA trace. Figure 10A,B shows that the thermal stability and char retention of the epoxy/microcapsule composite increased and the low temperature decomposition peak at 150 °C, associated with the microcapsule, has disappeared after the DMA test, indicating the occurrence of a reaction between HDI and epoxy to form a crosslinked stable structure.

It is shown that the combined percentage weight loss in transitions 1 and 2 after DMA testing, decreased to a great extent as compared to before DMA testing. This might indicate the rupture of microcapsules and the subsequent curing of HDI. The percentage of residue produced increases from a neat epoxy to an epoxy composite to an epoxy composite after DMA. In the epoxy composite before DMA, the encapsulated HDI evaporates completely but leaves behind char due to the PUF shell causing an increase in the percentage residue from neat epoxy. In the case of the epoxy composite after DMA, the residue increases further due to polyurea formation by the rupture of the shell followed by the release of HDI and a possible network formation.

### 3.6. Analysis of Microcapsules after Aging

**Figure 11:**
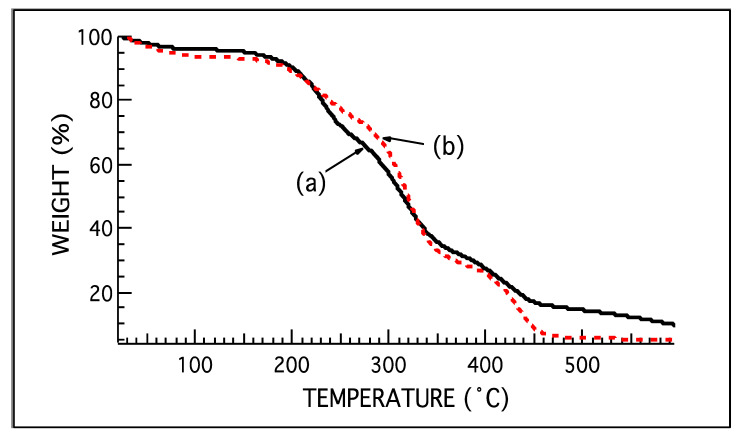
TGA of microcapsules with a water:oil ratio of 5.5:1 compared with TGA of the same microcapsules after aging for 4 months. (a) Percentage weight of microcapsules aged for 4 months; (b) Percentage weight of original microcapsules.

**Figure 12:**
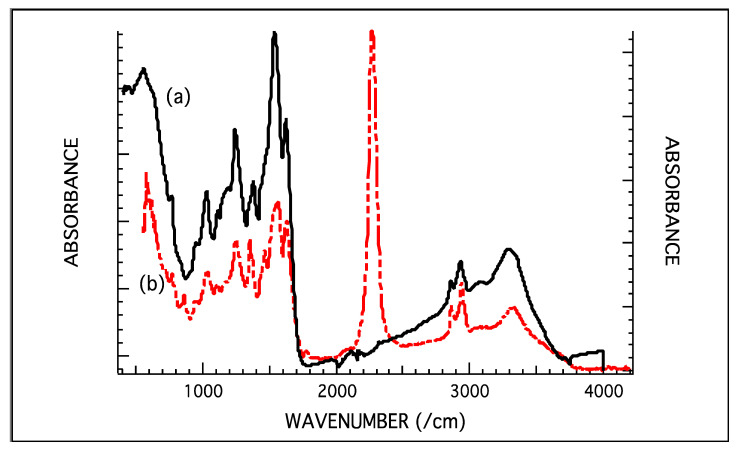
FTIR of microcapsules with a water:oil ratio of 5.5:1 compared with FTIR of the same microcapsules after aging for 4 months. (a) Microcapsules aged for 4 months; (b) Original microcapsules.

The data obtained from the TGA and FTIR analysis of the microcapsules made with a water-to-oil ratio of 5.5:1 after aging in ambient condition for four months are shown in Figure 11 and Figure 12. The TGA thermograph of the aged microcapsules (Figure 11), shows that the weight loss in transition 3 decreases from 46.36% to 35.86%, while the FTIR analysis shows that after aging, the NCO peak disappeared (Figure 12). These results lead to the inference that, over a period of time, an inside-out diffusion of HDI took place, forming an outer shell of polyurea, thus increasing the thickness of the shell, which is in agreement with reference [8]. The disappearance of the NCO peak in FTIR may be due to the fact that in ATR-FTIR analysis, the depth that can be analyzed is approximately 2 µm. The increase in shell thickness may have prevented the analysis of the core using ATR-FTIR.

**Figure 13:**
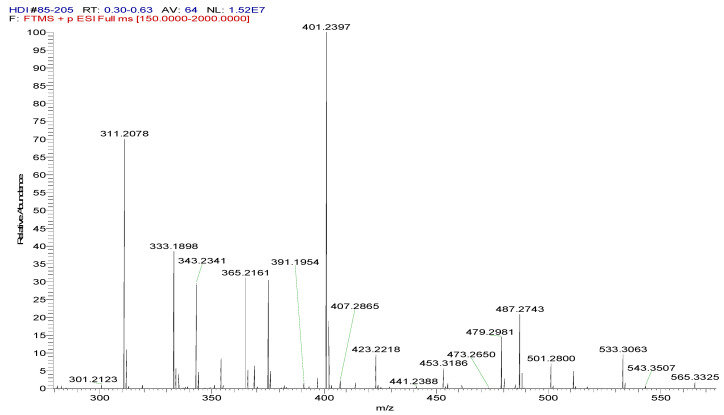
ESI mass spectrometry data for HDI.

**Figure 14:**
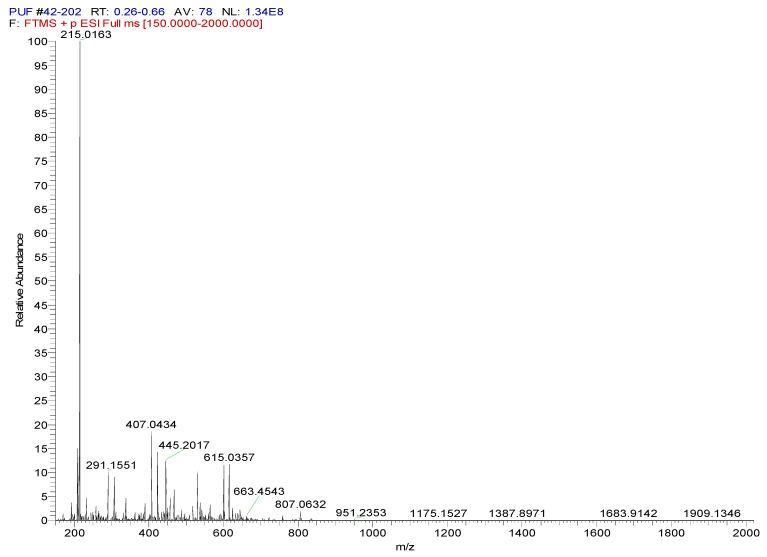
ESI mass spectrometry data for polyurea formaldehyde.

**Figure 15:**
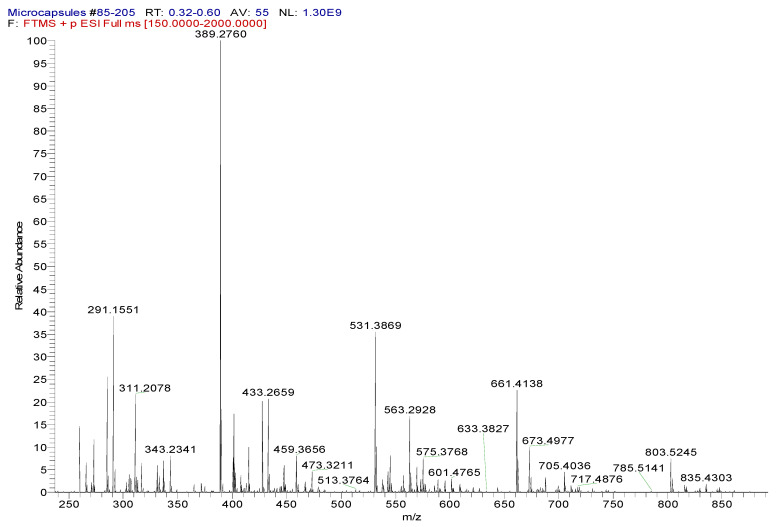
ESI mass spectrometry data for a microcapsule with a water-to-oil ratio of 5.5:1 aged for 4 months.

To further confirm the presence of HDI in the microcapsule, ESI mass spectrometry was performed. Mass spectrometry data was collected from the microcapsules, HDI, and freshly prepared PUF. PUF was prepared with the same formulation that was used for the microcapsule preparation with a twenty percent free formaldehyde content or a urea-to-formaldehyde content of 0.66. A process adopted from the procedure reported by Jin et al. [13] for the preparation of hollow microcapsules. Figure 13, Figure 14 and Figure 15 show ESI mass spectrometry graphs of HDI, PUF, and microcapsules with a water-to-oil ratio of 5.5:1, respectively. Peak 291.1551 from PUF and peak 311.2078 from HDI were also present in the ESI-MS graph for microcapsules. This confirmed the presence of PUF as well as HDI in the microcapsule.

## 4. Conclusions

1. HDI was successfully encapsulated in the PUF shell using a two-pot method. Water content was varied and different samples with water-to-oil ratios ranging from 4.5:1 to 6.5:1 were prepared.
2. The effect of increasing the water content in the oil-in-water emulsion was studied, and a maximum yield was obtained in the sample with a water-to-oil ratio of 5.5:1. The yield decreased as the water-to-oil ratio was decreased below a water-to-oil ratio of 5.5:1 and dropped drastically as the water-to-oil ratio was increased beyond 5.5:1.
3. Encapsulation of HDI was verified by optical microscopy and FTIR. The presence of diffraction rings in the optical images confirms the presence of a solid shell and liquid core. The presence of NCO at around 2200 cm^−1^ confirmed the presence of HDI in the system. The area under the NCO peak for all samples was measured using multipeak analysis from IGOR. The maximum area under the peak was found for a water-to-oil ratio of 5.5:1, and it decreased as the ratio decreased. The NCO peak was found to be absent in the sample with a water-to-oil ratio of 6.5:1.
4. Degradation characteristics of microcapsules were analyzed using TGA. The TGA graphs showed four distinct decomposition regions. The first decomposition region was attributed a low molecular weight species such as water and residual free formaldehyde, the second decomposition region was attributed to PUF, the third decomposition region was attributed to HDI evaporation, and the fourth decomposition region was attributed to polyurea degradation. Microcapsules with a water-to-oil ratio of 5.5:1 had the highest amount of weight loss in decomposition region 3. It was thus concluded that they had the highest encapsulation efficiency.
5. DMA was carried out to understand thermo-mechanical properties. Epoxy films containing 10-wt% of microcapsules were successfully prepared and tested. T_g_ of the system was successfully evaluated. A broad and intense tan∂ peak containing three peaks was found. These peaks related to the T_g_ of epoxy, T_g_ of microcapsules, and T_g_ of polyurea/epoxy.
6. Multi-run DMA analysis was performed on epoxy film containing microcapsules with a water-to-oil ratio of 5.5:1. The broad and intense tan∂ peak for the system disappears at the end of the third run, giving a single peak at 92 °C. By this, it was concluded that the rupture of the microcapsules with the epoxy coating caused a leakage of HDI within the film. This converted the heterogeneous epoxy/microcapsule system displaying three T_g_ into a homogeneous single-phase system displaying a single T_g_.
7. TGA was performed on the epoxy composite after DMA and the thermographs from TGA before DMA and after DMA were compared. Neat epoxy and epoxy composite before DMA showed three decomposition peaks, while the TGA trace after DMA showed three transitions, which was taken as an indication of polyurea presence. Another indication of polyurea presence was the increase in the percentage residue of epoxy composite after DMA from the neat epoxy and epoxy composite before DMA.
8. FTIR and TGA studies were carried out on the microcapsules after aging them for 4 months. It was found that the NCO peak in the microcapsules with a water-to-oil ratio of 5.5:1 completely disappeared. TGA analysis showed a decrease in the transition 3 region. It was thus concluded that HDI diffused out and formed a secondary shell layer, which increased its thickness. The increased shell made it difficult to detect the HDI core by ATR-FTIR.
9. ESI mass spectrometry was carried out on HDI, PUF, and microcapsules. The presence of peaks pertaining to HDI and PUF in the mass spectrometry of microcapsules confirmed the presence of PUF and HDI.

## Figures and Tables

**Table 1:** Thermal transition temperature of materials.

S/N	Material	Transition Temperature, T_g_ (°C)
1	Epoxy	35–37
2	Microcapsule	59
3	Polyurea	92–93
4	Crosslinked epoxy/polyurea networks	123

## Data Availability

Data presented in this study are available on request from the corresponding author.
